# Febuxostat Attenuates Renal Damage besides Exerting Hypouricemic Effect in Streptozotocin-Induced Diabetic Rats

**DOI:** 10.1155/2017/2739539

**Published:** 2017-04-19

**Authors:** Jianmin Ran, Gang Xu, Huixuan Ma, Hailing Xu, Yan Liu, Rongshao Tan, Ping Zhu, Jun Song, Gancheng Lao

**Affiliations:** ^1^Department of Endocrinology, Guangzhou Red Cross Hospital, Medical School of Jinan University, No. 396 Tong Fu Zhong Road, Guangzhou, China; ^2^Guangzhou Institute of Disease-Oriented Nutritional Research, Guangzhou Red Cross Hospital, Medical School of Jinan University, No. 396 Tong Fu Zhong Road, Guangzhou, China; ^3^Department of Nephrology, Guangzhou Red Cross Hospital, Medical School of Jinan University, No. 396 Tong Fu Zhong Road, Guangzhou, China; ^4^Southern Medical University, No. 1023-1063 Southern Sha Tai Road, Guangzhou, China

## Abstract

*Aim*. In this study, we aimed to investigate the effects of febuxostat, a novel inhibitor of xanthine oxidase (XO), on renal damage in streptozotocin- (STZ-) induced diabetic rats.* Methods*. Diabetes was induced by the intraperitoneal injection of STZ in male Sprague-Dawley rats. Sham-injected rats served as controls. The control and diabetic rats were treated with and without febuxostat for 8 weeks, respectively. Fasting blood and 24-h urine samples were collected every 4 weeks. Rat livers were extracted for detecting gene expression, content, and bioactivity of XO.* Results*. Diabetic rats showed significantly increased serum uric acid (SUA), serum creatinine (SCr), and urea nitrogen (BUN) levels. Daily urinary albumin (UAE), uric acid (UUA), and creatinine (UCr) excretion were also significantly increased in these rats. In diabetic rats, at week 8, febuxostat decreased SUA by 18.9%, while UAA was increased by 52.0%. However, UCr and urinary urea nitrogen (UUN) levels remained unchanged, while SCr and BUN levels decreased by >30% in these rats. Although hepatic gene expression, content, and activity of XO increased significantly in diabetic rats, febuxostat only slightly decreased its content.* Conclusions*. Febuxostat significantly attenuated renal damage in STZ-induced diabetic rats in addition to exerting hypouricemic effect.

## 1. Introduction

Diabetic kidney disease (DKD) is the leading cause of end stage renal failure (ESRD) worldwide [1]. For many years, several mechanisms including renal hemodynamic alterations, renin-angiotensin-aldosterone system (RAAS) activation, inflammatory pathways, and reactive oxygen species (ROS) were widely studied in DKD, and various corresponding therapeutic agents have been developed [2]. However, DKD outcomes following administration of these therapeutic agents offered no promising improvement [1]. The underlying mechanisms and interventional targets of DKD should be essentially explored.

Several cohort and cross-sectional studies definitively established the relationship between hyperuricemia and the progress of DKD in either type 1 or type 2 diabetes [3–5]. Several clinical studies using hypouricemic agents such as allopurinol showed positive outcomes such as improving renal damage and postponing renal failure in patients with either diabetes or chronic kidney disease (CKD) [6]. Febuxostat (Fx) is a recently developed xanthine oxidase (XO) inhibitor, which has been definitively proved to be effective and safe for gout treatment [7]. XO is an enzyme that generates ROS by catalyzing the oxidation of hypoxanthine to xanthine and xanthine to uric acid. Some of the renal protective effects of Fx were clarified in animal models with diabetes, such as db/db mice [8] and diabetic Zucker rats [9]. Despite these promising data, we noticed that the plasma uric acid (UA) levels in most studies were normal or even low because of the degradation of uricase, an enzyme that converts uric acid to allantoin, which is much more soluble than uric acid [10]. Alterations in UA metabolism were also seldom discussed in these papers. Suitable animal models characterized by both diabetes and hyperuricemia should be explored for researches in this field.

In our previous studies [11] on streptozotocin- (STZ-) induced diabetic rats, we found that serum UA concentration was significantly and permanently increased, which was accompanied by abnormalities in renal function, including increased serum creatinine (Scr) and albuminuria; enlarged glomeruli and tubular hyalinization were also prominent in these rats. Similar increase in UA concentration was seen in STZ-induced diabetic rats in other studies [12, 13]. Therefore, this rat model is more appropriate for researches on hyperuricemia in diabetic conditions. In the present study, we investigated the effects of Fx on renal injury in STZ-induced diabetic rats with the aim to search novel therapeutic method for DKD.

## 2. Materials and Methods

### 2.1. Animal Preparation

**Figure 1:**
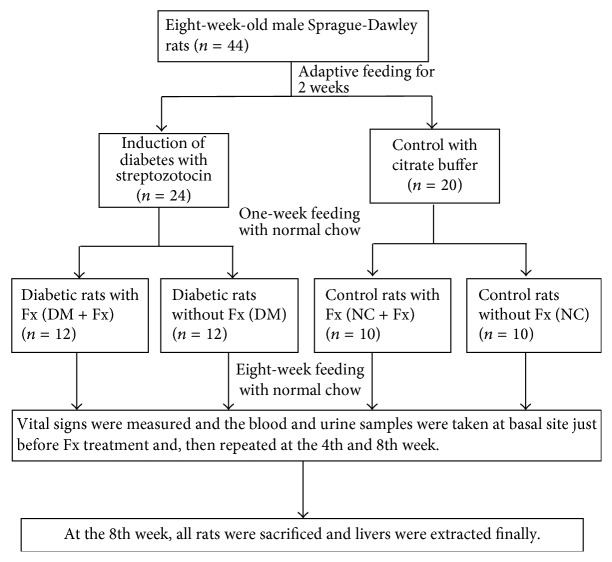
Schematic diagram of the animal experiment protocol. Fx: febuxostat. NC: the normal control group without Fx treatment; NC + Fx: the normal group with Fx treatment; DM: diabetic mellitus group without Fx treatment; DM + Fx: diabetic mellitus group with Fx treatment.

The overall animal experiment protocol is shown in Figure 1. Eight-week-old male Sprague-Dawley rats (Guangdong Medical Laboratory Animal Center, Foshan, China) weighing 200–220 g were adopted for this study. All rats were collectively housed (2 rats per cage) and fed with standard rat chow for 2 weeks. For diabetes induction, the rats were intraperitoneally injected with STZ (dissolved in 50 mM citrate, pH = 4.2, Sigma, St Louis, USA) at a single dose of 65 mg/kg. Twenty-four rats with random blood glucose levels > 16.7 mmol/L at three different times were selected for the experiments. Twenty rats that served as controls were intraperitoneally injected with the same volume of citrate buffer.

### 2.2. Fx Treatment and Animal Experiments

After successful induction of diabetes for 2 weeks, the experimental diabetic and control rats were treated with Fx (Melone Pharmaceutical Co., Ltd, Dalian, China), which was dissolved in 0.5% carboxymethylcellulose sodium (CMC-Na, Fu Chen Chemical Reagents Factory, Tianjin, China), at a dose of 5 mg/kg/d via daily gavage for 8 weeks (Figure 1). The control rats were treated only with the same volume of CMC-Na. The rats were divided into 4 groups during this intervention period as follows: diabetic rats with (DM + Fx, *n* = 12) and without (DM, *n* = 12) Fx treatment, as well as normal control rats with (NC + Fx, *n* = 10) and without (NC, *n* = 10) Fx treatment.

Vital signs, including systolic blood pressure (SBP), diastolic blood pressure (DBP), and heart rate (HR) were recorded in fully conscious rats by using indirect tail-cuff equipment (LE5002, Harvard Apparatus, USA). After prewarming the rats for 20 min on a 37°C plate, the SBP, DBP, and HR of each rat were recorded.

Blood and urine samples were collected every 4 weeks at the baseline and at weeks 4 and 8, respectively. For urine sampling, the rats were individually housed in metabolic cages for 24 h; then, all urine samples were collected and volumetrically estimated. All rats were sacrificed after 8 weeks of Fx treatment, and the livers were removed for histologic, enzymatic, and genetic assays.

During the whole experiment, all rats were allowed free access to standard rat chow and water, and the room light was rotated at a 12-h light-dark cycle. On the morning of the experiment, foods were withdrawn 12 h before each operation. All animal experimental procedures were approved by the Ethnic Committee of Guangzhou Red Cross Hospital.

### 2.3. Biochemical Assays

The serum concentrations of glucose, triglyceride (TG), total cholesterol (TC), uric acid (SUA), urea nitrogen (BUN), and SCr were measured using the corresponding commercial kits on an automatic biochemical machine (ECHO, ECHO, Italy). The 24-h urine samples were collected and quantified. Urinary uric acid (UUA), urinary urea nitrogen (UUN), and urinary creatinine (UCr) were detected by the same automatic machine. Urinary albumin was determined by the standard bromocresol green method, and the 24 h amount of urinary albumin excretion (UAE) was then calculated.

### 2.4. Hepatic Content and Activity of XO

The hepatic content of XO was measured according to the previously described method [14]. Liver tissues weighing 0.25 g were mixed with 9 times the volume of purified water and homogenized. This mixture was centrifuged for 10 min at 3000 rpm, and the supernatant was separated. The XO concentration in the supernatant was measured by using the corresponding commercial ELISA kits (Huamei Bioengineer Ltd. Co, Wuhan, China).

For the measurement of hepatic XO activity, we determined the total protein content of the homogenate according to the Coomassie brilliant blue method [15] using a commercial reagent kit (Nanjing Jiancheng Bioengineering Institute, Nanjing, China). Substrate and buffers were added in the test and control reaction system (Nanjing Jiancheng Bioengineering Institute, Nanjing, China), respectively. The absorbance was measured at 530 nm after 20 min incubation at 37°C. Hepatic XO activity was calculated according to the absorbance difference and expressed as U/g protein.

### 2.5. Gene Expression

Hepatic gene expression of XO, the key enzyme for UA formation, was determined by real-time polymerase chain reaction (RT-PCR). Frozen tissues were homogenized and total RNA was extracted using a TRIzol kit (Invitrogen, CA, USA). RNA quality and quantity were assessed by automated capillary gel electrophoresis on a Bioanalyzer 2100 with RNA Nano LabChips (Agilent Technology, Tokyo, Japan). Then, total RNA (1 *μ*g) was reversely transcribed using a cDNA synthesis kit (Promega, CA, USA) with random primers in a 20 *μ*L PCR system according to the manufacturer's protocol. Quantitative PCR was performed by SYBR Green PCR Master Mix (Toyobo, Osaka, Japan) and ABI PRISM 7500 Sequence Detection System (Applied Biosystems Inc., CA, USA). Thermal cycling was carried out at 95°C for 15 min, followed by 40 cycles at 95°C for 15 s, 60°C for 15 s, and 72°C for 32 s. We used 18S rRNA as a housekeeping gene in RT-PCR. The specific primers were selected as follows: XO forward: 5′-GACAGGGTGTTTATGAAGCA-3′, XO reverse: 5′-AACTCACTGCGCTCGTATAG-3′; 18S rRNA forward: 5′-CCTGGATACCGCAGCTAGGA-3′, 18S rRNA reverse: 5′-GCGGCGCAATACGAATGCCCC-3′.

### 2.6. Statistical Analysis

The results are expressed as mean ± SD. One-way analysis of variance (ANOVA) and Mann–Whitney *U* test were selected for comparisons of differences between means and nonnormally distributed data differences, respectively. Statistical difference was accepted at *P* < 0.05.

## 3. Results

### 3.1. General Characteristics

**Figure 2:**
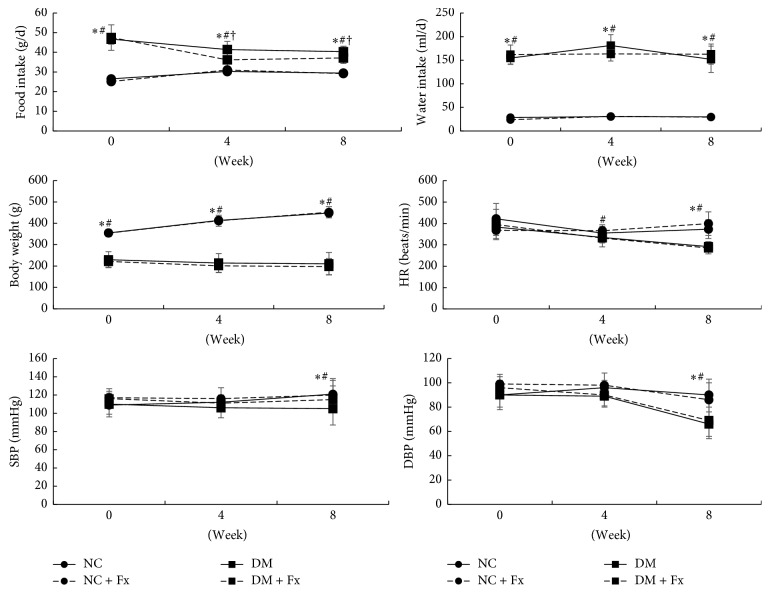
Time course of food intake, water intake, body weight, and main vital signs during the experiment after induction of diabetes. Heart rate (HR), systolic blood pressure (SBP), and diastolic blood pressure (DBP) were recorded by an indirect tail-cuff method. The circle with solid and dashed lines represents NC and NC + Fx groups, respectively, while the block symbol with solid and dashed lines shows data of DM and DM + Fx groups, respectively. Data are expressed as mean ± SD. Statistical significance (*P* < 0.05) was labeled as *∗*, #, and † correspondingly, when the DM + Fx group was compared with NC, NC + Fx, and DM groups.

Although the diabetic rats were more polyphagous and polydipsic than normal rats after induction of diabetes, Fx treatment reduced the daily food intake in diabetic rats at the 4th and 8th weeks (Figure 2, both *P* < 0.05). The daily water intake was not affected by Fx treatment in any rats. Diabetic rats lost their weight significantly, while the body weight of normal rats increased continuously during the whole experiment. In case of vital signs, the diabetic rats showed lower HR than normal rats at the 8th week (*P* < 0.05); SBP and DBP decreased in diabetic rats at this time point (*P* < 0.05). However, Fx did not exert any effects on these vital signs either in diabetic or normal rats.

### 3.2. Blood and Urine Biochemistry

**Figure 3:**
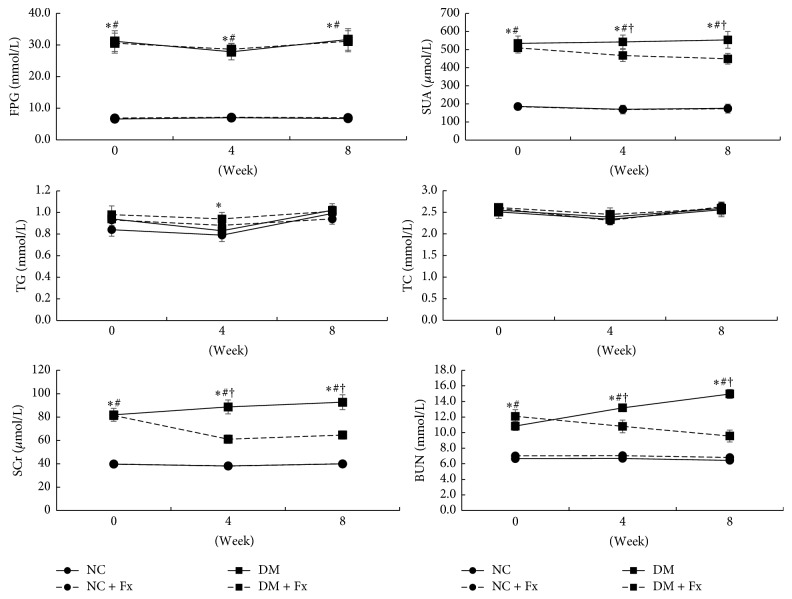
Time course of blood biochemical indices among the four rat groups. The circle with solid and dashed lines represents NC and NC + Fx groups, respectively, while the block symbol with solid and dashed lines shows data of DM and DM + Fx groups, respectively. Data are expressed as mean ± SD. Statistical significance (*P* < 0.05) was labeled as *∗*, #, and † correspondingly, when the DM + Fx group was compared with NC, NC + Fx, and DM groups.

The blood biochemical profile throughout the experiment is shown in Figure 3. After successful induction of diabetes, the fasting plasma glucose (FPG) in diabetic rats was maintained at very high levels during the experiment compared to that in normal rats (all *P* < 0.05). NC + Fx rats showed slightly higher plasma TG than the NC control group (*P* < 0.05), while the plasma TC level was comparable among all rat groups. In both diabetic and normal rats, Fx treatment did not alter the plasma glucose and lipid levels. SUA, SCr, and BUN levels increased significantly (all *P* < 0.05) in diabetic rats at the baseline. In diabetic rats, Fx treatment slightly decreased SUA by 18.9% at the 8th week (*P* < 0.05), while both SCr and BUN were significantly decreased to around 30.0% (both *P* < 0.05). SUA, SCr, and BUN were not affected by Fx treatment in normal rats (all *P* > 0.05).

**Figure 4:**
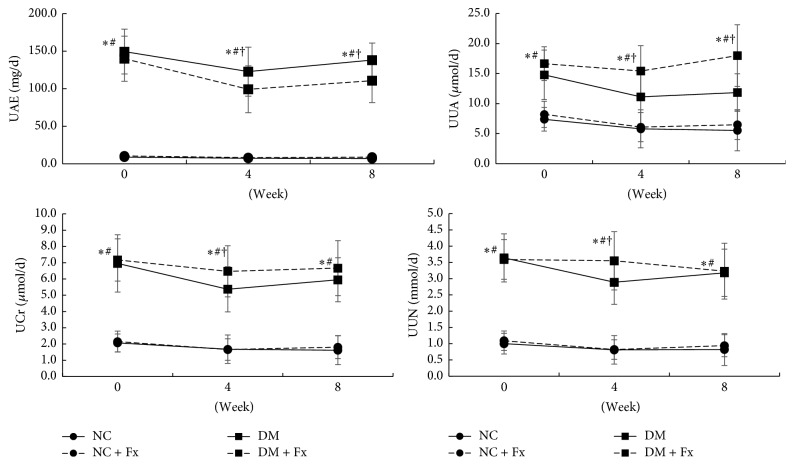
Time course of daily urinary excretions among the four rat groups. The circle with solid and dashed lines represents NC and NC + Fx groups, respectively, while the block symbol with solid and dashed lines shows data of DM and DM + Fx groups, respectively. Data are expressed as mean ± SD. Statistical significance (*P* < 0.05) was labeled as *∗*, #, and † correspondingly, when the DM + Fx group was compared with NC, NC + Fx, and DM groups.

Daily urinary excretions are depicted in Figure 4. UAE (at week 0, 149.42 ± 29.85 mg/d, 140.04 ± 30.11 mg/d, 8.89 ± 1.96 mg/d, and 10.52 ± 2.39 mg/d for DM, DM + Fx, NC, and NC + Fx group, resp.), UUA, UCr, and UUN remarkably increased in the diabetic rats (all *P* < 0.05 when DM was compared with NC group). Fx treatment significantly decreased the daily UAE level at the 4th (122.84 ± 32.65 mg/d for DM and 99.25 ± 31.25 mg/d for DM + Fx, *P* < 0.05) and 8th week (138.21 ± 22.57 mg/d for DM and 110.84 ± 29.18 mg/d for DM + Fx, *P* < 0.05) in diabetic rats. Notably, in diabetic rats, Fx treatment significantly increased UUA at the 4th and 8th weeks (both *P* < 0.05). In particular, at the 8th week, the daily UUA was significantly increased to 52.0% following Fx treatment. UCr and UUN were also increased after Fx treatment at the 4th week (both *P* < 0.05), but they were comparable at the 8th week (both *P* > 0.05). Fx did not change the aforementioned daily urinary excretions in normal rats during the experiment.

### 3.3. Hepatic Content, Activity, and Gene Expression of XO

**Figure 5:**
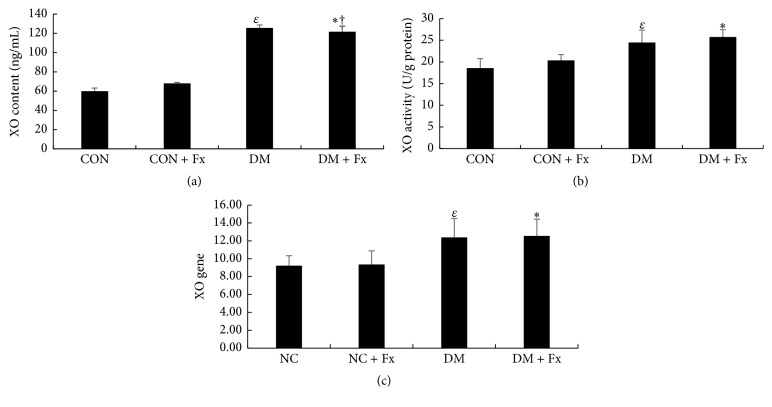
Hepatic content, activity, and gene expression of xanthine oxidase (XO). Hepatic content of XO (a) was expressed as its concentration in the supernatant of liver extract. Hepatic XO activity (b) was calculated according to the XO protein ratio and expressed as U/g protein. Relative gene expression (c) was determined by RT-PCR and shown in the graph as the amounts of initial template. Data are expressed as mean ± SD. ^*ε*^*P* < 0.05 when the DM group was compared with CON or CON + Fx group, ^*∗*^*P* < 0.05 when DM + Fx group was compared with CON or CON + Fx, and † when compared with DM group, respectively.

The hepatic content of XO (125.59 ± 3.04 ng/mL for the DM group versus 59.94 ± 3.23 ng/mL for the CON group, *P* < 0.05) increased significantly in diabetic rats (Figure 5(a)). In addition, the enzymatic activity of XO (Figure 5(b)) increased significantly in diabetic rats compared to that in normal control rats (24.42 ± 2.95 U/g protein for the DM group versus 18.60 ± 2.16 U/g protein for the CON group, *P* < 0.05). Gene expression of XO showed the same trend as that of hepatic content and enzymatic activity (Figure 5(c)).

Fx treatment slightly decreased the hepatic XO content in diabetic rats (Figure 5(a), *P* < 0.05). Nevertheless, the treatment exerted no effects on hepatic enzymatic activity and gene expression of XO in either diabetic or normal rats (Figures 5(b) and 5(c), all *P* > 0.05).

## 4. Discussion

Similar to our previous study [11], we found that STZ-induced diabetic rats developed high levels of SUA and renal damage, marked by elevated serum BUN, SCr, and daily UAE. Fx, a specific XO inhibitor, significantly reduced SUA and attenuated renal function without affecting the blood glucose, blood pressure, and lipid profile. These findings indicated that hyperuricemia and its related pathological processes could be an important and direct mechanism underlying renal damage in STZ-induced diabetic rats. Correspondingly, all therapies focusing on UA metabolism may retard the progression of diabetic renal injury [16].

It remains unclear how UA directly facilitates renal damage in diabetic patients and various diabetic animal models [17]. It is well known that RAAS triggers hemodynamic changes and inflammatory attacks; therefore, it plays pivotal roles in DKD [18]. In vivo studies [19, 20] have demonstrated that UA may promote RAAS activity in CKD animal models. Several researches have reported links between UA metabolism and other proinflammatory pathways [21, 22]. Among them, the convincing one is that UA, as crystals causing cellular necrosis, can activate the inflammasome NLRP3, which consequently induces caspase-1 and its downstream cytokines including IL-1*β* and IL-18 [23, 24]. The latter two cytokines have been proved to be potentially expressed on tubular epithelial cells and may closely relate to UA-induced interstitial damage [25].

The present study showed that Fx significantly reduced SUA by 18% and attenuated renal damage. This result is consistent with that of several other animal and clinical studies. In diabetic db/db mice [26], Kosugi et al. found that tubulointerstitial injury is significantly attenuated by treatment with allopurinol, another XO inhibitor, for 8 weeks. Following Fx treatment, normalization of SUA and improvements in renal injury were also achieved in several diabetic models such as db/db mice [8, 27], Zucker diabetic rats [9], and STZ-induced diabetic rats [28]. Large-scale clinical trials on the effects of lowering SUA on DKD progression are still scarce [29]. In a previous study in type 2 diabetic patients with DKD, daily UAE was significantly reduced after a 4-month intervention with allopurinol [30]. Recently, another study [31] showed that 3-year treatment with allopurinol in type 2 diabetic patients with asymptomatic hyperuricemia decreased UAE and SCr, while the glomerular filtration rate was increased. In the present study, daily UUN, UCr, and UUA increasing significantly after Fx treatment might be attributed to improved glomerular filtration; we regarded this as a novel finding if compared with other animal studies.

Compared with the above-mentioned animal researches, the most noticeable thing in our experiment is that Fx treatment decreased SUA to approximately 18% in diabetic rats, but in normal rats SUA was unchanged at the 8th week. Thus, SUA in DM + Fx group was still significantly higher than that in both NC and NC + Fx groups. According to previous studies, Fx at 5 mg/kg is a moderate dose for rats and mice [32]; however, we did not observe complete normalization of SUA when compared to that in few other studies involving SUA [33]. One reason for this might result from the relatively short-term intervention of Fx (only 8 weeks). Second, we speculate that STZ might aggravate UA metabolism by directly suppressing uricase to some extent, instead of promoting in vivo XO activity [34]. Significantly increased hepatic XO gene expression, content, and activity in diabetic rats were observed in our research, which were similar to those observed in several studies using other diabetic models, such as Zucker diabetic rats [9], Otsuka Long-Evans Tokushima fatty rats [35], and db/db mice [8]. We may conclude that diabetes itself rather than STZ activates XO, thereby promoting UA production in these rats [36]. This hypothesis can illustrate why Fx only slightly decreases SUA in STZ-induced diabetic rats when no hypoglycemic treatment is provided. Further studies are needed to elucidate the relationship between STZ and UA metabolism.

Despite the slight reduction of SUA, remarkable renal protective effects were observed in the diabetic rats. Daily UAE significantly decreased, and, more importantly, serum BUN and SCr decreased by more than 30% in these rats. Fx treatment should specifically target XO and this can inhibit in vivo UA production [9]. Hepatic content, activity, and gene expression were measured in our experiment. All these parameters were significantly increased in diabetic rats in agreement with the findings of other studies [8, 9, 35]. Nevertheless, exceptional results were observed in this study, including unchanged XO gene expression and activity and a slight decrease in hepatic XO content after Fx treatment in diabetic rats. Gene expression and activity of XO were unchanged which may result from the upregulating effects after the competitive inhibition by Fx. But anyway, Fx decreased SUA slightly and exerted minor effects on hepatic XO content. Simultaneously, Fx treatment did not make significant changes in body weight, blood pressure, and metabolic indices including blood glucose and lipid levels. These data throw light on mechanisms other than hypouricemic effect that may underlie the renal protective effects of Fx.

Several other researches explored the mechanisms involved in the attenuation of renal injury by Fx treatment. Sánchez-Lozada et al. found that Fx significantly reduced glomerular pressure and renal vasoconstriction in fructose-induced metabolic syndrome [33] and oxonic acid-induced hyperuricemic rat models [37]. Moreover, in STZ-induced diabetic rat models, Lee et al. [28] showed that Fx prevents renal damage mainly by ameliorating the inflammatory factors and oxidative stress, owing to its inhibitory effects on XO. In a recent study in Zucker diabetic rats, Komers et al. [9] examined the effect of Fx on oxidative stress. The simultaneous inhibition of profibrotic signaling by Fx might be another pivotal mechanism. We did not perform further experiments on glomerular structure and interstitial changes in our study. However, it is well known [38] that UA preferentially induces tubular damage. In our previous study, feeding the same diabetic rats with low protein diets decreased SUA and attenuated tubular injuries. In the present study, we noticed that daily UAE was increased after Fx treatment in diabetic rats, which could not be appropriately explained by its direct pharmaceutical actions [39]. Unknown but important mechanisms of Fx on renal tubular damage should be investigated in future researches.

This study has several limitations. First, the morphologic alterations of glomerular and interstitial area after Fx treatment should be studied in a long-term experiment. Second, the renal hemodynamic parameters were not included in the study design, and these alterations may partly be responsible for the improvement of renal function in diabetic rats. Researches on the direct effects of UA on renal damage in diabetes have been initiated and more compromised data will be provided in the future.

## 5. Conclusions

Fx attenuated renal damage without any significant influence on glucose, blood pressure, and lipid levels in STZ-induced diabetic and hyperuricemic rats. The mild hypouricemic effects and actions of Fx on XO pave the way for researchers to explore other underlying mechanisms, especially in the tubules, besides its traditional targets.
